# Supplementary material for: Genetic comparisons of interleukin-17 reveal a framework for complex signaling evolution
Source: bioRxiv. 2026 Apr 14:2026.04.13.718218. Preprint. [Version 1] doi: 10.64898/2026.04.13.718218 (PMC13105070; doi:10.64898/2026.04.13.718218)
Supplement: Supplement 3 [file media-3.zip › Supplemental files for paper/ERC/IL17xIL17_comparisons/IL17RD_IL25.pdf]

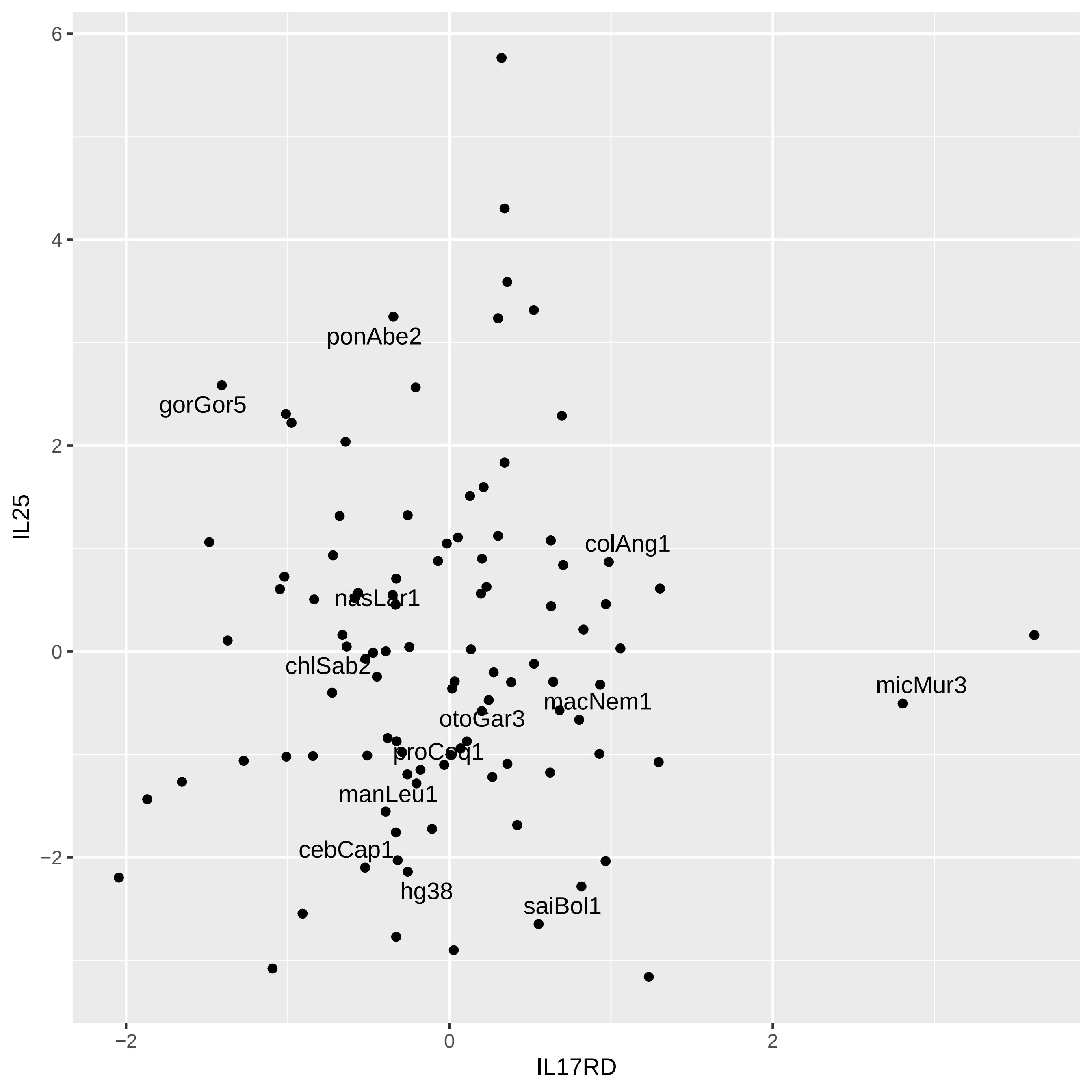

IL25
IL17RD
6
4
2
0
−2
−2
0
2
ponAbe2
gorGor5
colAng1
nasLar1
chlSab2
micMur3
macNem1
otoGar3
proCoq1
manLeu1
cebCap1
hg38
saiBol1
